# Isolation and characterization of *Yersinia enterocolitica* from foods in Apulia and Basilicata regions (Italy) by conventional and modern methods

**DOI:** 10.1371/journal.pone.0268706

**Published:** 2022-07-13

**Authors:** Maria Emanuela Mancini, Matteo Beverelli, Adelia Donatiello, Antonella Didonna, Luigi Dattoli, Simona Faleo, Gilda Occhiochiuso, Domenico Galante, Valeria Rondinone, Laura Del Sambro, Angelica Bianco, Angela Miccolupo, Elisa Goffredo

**Affiliations:** Istituto Zooprofilattico Sperimentale della Puglia e della Basilicata, Foggia, Italy; Fisheries and Oceans Canada, CANADA

## Abstract

Yersiniosis is the third most reported food-borne zoonosis in Europe. The aim of the present study was to perform the search for *Yersinia enterocolitica* in food samples collected from Apulia and Basilicata regions (Southern Italy) and to characterize any isolates by classical and modern analytical methods. A total of 130 samples were analyzed between July 2018 and July 2019: most of them were raw milk and dairy products made from it. Furthermore, 8 out of 130 samples were individual milk samples collected from bovines reared in a *Brucella*-free farm which showed false positive serological reaction for brucellosis due to the presence of pathogenic *Y*. *enterocolitica* O:9 biotype 2 in faeces. The Real Time PCR targeting the *ail* gene and the culture method were performed to detect pathogenic *Y*. *enterocolitica*. Isolates were subjected to API 20E (Biomerieux) and MALDI-TOF MS (Matrix Assisted Laser Desorption Ionization Time-of-Flight) for species identification. All samples were negative for the *ail* gene. The culture method allowed to isolate suspicious colonies from 28 samples. The API 20E system and the MALDI-TOF MS technique identified 20 *Y*. *enterocolitica* and 1 *Y*. *intermedia* in a concordant way. The remaining 7 strains were all identified as *Y*. *enterocolitica* by the API 20E system, while the MALDI-TOF MS recognized 4 *Y*. *intermedia*, 1 *Y*. *bercovieri* and 2 *Y*. *massiliensis*. Genotypic characterization of the discordant strains was performed by rMLST and it confirmed the MALDI-TOF MS’ results. Only non-pathogenic *Y*. *enterocolitica* biotype 1A strains were found, although with a non-negligible prevalence (P = 0.15 with CI _95%_ = ± 0.06). This study indicates a poor circulation of pathogenic *Y*. *enterocolitica* in food products made and marketed in the investigated areas. However, the small number of samples, insufficient for some food categories such as meat and vegetable, does not allow to exclude the presence of pathogenic strains at all.

## Introduction

The genus *Yersinia* spp. belongs to the *Enterobactericeae* family and includes 18 species, three of which are pathogenic for humans and animals: *Y*. *enterocolitica* and *Y*. *pseudotubercolosis*, responsible for gastroenteritis, and *Y*. *pestis* x [1]. Eleven of the 18 recognized species were initially included in the group of *Y*. *enterocolitica*-like microorganisms and then recognized as species in their own right: *Y*. *aldovae*, *Y*. *bercovieri*, *Y*. *frederiksenii*, *Y*. *intermedia*, *Y*. *kristensenii*, *Y*. *mollaretii*, *Y*. *rohdei*, *Y*. *ruckeri*, *Y*. *alecksiciae*, *Y*. *similise Y*. *massiliensis* [2, 3]. Most of them can be distinguished on the basis of their main phenotypic characteristics, but this is not true for some species such as *Y*. *alecksiciae* which cannot be differentiated from *Y*. *kristensenii* [4]. Moreover, it seems that some strains belonging to the *Y*. *enterocolitica*-like species may be responsible for human disease [5]. *Yersinia enterocolitica* is Gram negative, rod-shaped, 0.5–0.8 μm wide and 1–3 μm long, facultative anaerobic, not capsulated, non-sporogenous, motile by peritrichous flagella at temperatures between 20°C and 30°C (optimum 25°C) and immobile at 37°C [6].

In general, *Y*. *enterocolitica* can grow over a wide range of temperature (0°C—45°C), with temperature optimum of 25–35°C [7]. It is a psychrophilic microorganism and grows well at temperatures below 4°C [8, 9], can proliferate in meat and milk at temperatures even below 0°C [10], resists freezing and is capable of long-term survival in frozen foods. Its ability to grow at refrigeration temperatures in vacuum packaged or modified atmosphere packaged food products [11, 12] is extremely important for food safety. Conversely, it is sensitive to heat: the heat treatment of milk or meat products at 60°C for 1–3 minutes inactivates it effectively [10]. Moreover, it does not survive pasteurization, although there are reports on its isolation from pasteurized milk, but this could be due to ineffective pasteurization and/or post-process contamination [13, 14]; in any case, no heat-resistant strains have been reported [15]. However, thanks to the absence of competitive microflora, pasteurized milk offers a favorable environment for *Y*. *enterocolitica* growth; even when a low initial contamination level occurs, this bacterium can multiply over hours or days until it reaches the infectious dose, depending on the storage temperature [16].

*Yersinia enterocolitica* is divided into two subspecies, *Y*. *enterocolitica* subsp. *enterocolitica* and *Y*. *enterocolitica* subsp. *palaearctic*, on the basis of two different 16S rRNA gene type, as proposed by Neubauer *et al*. [17]. *Yersinia enterocolitica* is classified into 6 biotypes (1A, 1B, 2, 3, 4, 5) [18] based on its ability to ferment different substrates. The biotypes 1B, 2, 3, 4 and 5 are frequently isolated in human infections. Biotype 1A strains are ubiquitous and have been found in a wide variety of environments (soil and water sources), food (vegetables and animal products) and animals (mammals, birds, fish, insects and frogs) [19]. They are usually considered non-pathogenic because they do not possess either the plasmid (pYV) which encodes virulence factors including Yersinia adhesin A (YadA) and Ysc-Yop type III secretion system (TTSS) and most of the chromosomal virulence genes such as *ail*, *myfA*, *ystA* and the high pathogenicity island-associated iron acquisition system [19]. However, biotype 1A strains may occasionally carry the *ystA*, *myfA* and *ail* genes [19, 20]. Furthermore, some 1A strains have been isolated from people with gastrointestinal disease and are capable of producing the thermostable enterotoxin encoded by the *ystB* gene; they have also been involved in extraintestinal infections, as well as in nosocomial and food-borne outbreaks [19].

More than 70 serotypes are recognized [21], on the basis of the characteristics of lipopolysaccharide O; among them, O: 3, O: 8, O: 9, and O: 5,27 are the serotypes most involved in human infections [22, 23]. In particular, the bioserotype most responsible for human cases in Europe is 4/O: 3, followed by 2/O: 9 and 2/O: 5.27 [24]. Furthermore, *Y*. *enterocolitica* O:9 is often associated with false-positive serological reactions in the diagnosis of brucellosis in ruminants [25–27]. In fact, its O-antigen shows a high similarity to the smooth lipopolysaccharide O-antigen of Brucella which is used in diagnostic tests recommended by the OIE [28, 29].

Pigs are considered the main reservoir of human pathogenic *Y*. *enterocolitica* [30, 31] and they are usually referred to as asymptomatic carriers [32]. The main localization sites are oral cavity, tongue, tonsils, lymph nodes and intestine and the microorganism is excreted via faeces [9, 30, 33]. Therefore, meat contamination with *Y*. *enterocolitica* can occur during slaughter and subsequent handling: the meat cuts closest to head and sternum are mainly exposed [34].

Food is the main source of human infection and food contamination can be primary or subsequent to contact with contaminated surfaces and equipment. The occurrence of *Y*. *enterocolitica* is reported not only in raw or undercooked meat (pork, chicken, beef and sheep) but also in raw or pasteurized milk and dairy products made from it (e. g. soft cheeses), fish products (fish, raw oysters, shrimps, crabs), fruits, vegetables (e.g. soy sprouts), tofu and drinking water [15, 16, 19, 34–36]. Although fruit and vegetables are considered possible sources of *Y*. *enterocolitica* [37, 38], there are few studies until now [32]. On the contrary, a lot of studies report the presence of pathogenic *Y*. *enterocolitica* in samples of raw milk collected from various animal species, as consequence of faecal contamination during milking [13, 14, 39–43].

Human-to-human transmission is rare but cases of food contamination by infected food handler as well as nosocomial infections are reported; the microorganism can also be transmitted through infected blood, and it was one of the first recognized causes of post-transfusion sepsis [19].

Yersiniosis is the third most reported food-borne zoonosis in Europe, with 6823 confirmed human cases in 2017 [24]. The main clinical manifestation of yersiniosis is a gastroenteritis, often in a self-limiting form: generally, the symptoms disappear within 1–3 weeks [44]. Enterocolitis is mainly observed in children, while older children and adolescents manifest usually a pseudo-appendicitis syndrome [45]. The infectious dose is 10^8^−10^9^ cells [34], the incubation time is about 3–7 days, but it can last between 1 and 11 days [44].

Species-specific identification and subtyping of *Yersinia enterocolitica* have traditionally been based on biochemical methods and, in recent decades, on biomolecular ones [46]: a gene target generally used for taxonomic purposes is the 16S rRNA gene [17]. PCR methods targeting chromosomal virulence genes such as the *ail* gene are more frequently used for detection of pathogenic *Y*. *enterocolitica* strains [47] because of possible plasmid loss. The Matrix-assisted laser desorption/ionisation-time of flight mass spectrometry (MALDI-TOF MS) has been recently introduced in microbiology laboratories as a rapid, accurate and economical method for the identification of bacteria, mycobacteria, yeasts and fungi [48]. It has also been applied for the identification of *Yersinia* species [49–51].

The aim of the current study was to acquire information on the circulation of *Y*. *enterocolitica* in foods of animal and vegetal origin produced and/or marketed in Apulia and Basilicata regions (Southern Italy) in order to improve the knowledge on trends and potential sources of this pathogen along the food chain. Furthermore, the European Food Safety Authority reported the lack of data provided by Member States about the findings of *Y*. *enterocolitica* in food and animals in 2017 [24]. The intent of this research was also to compare two different methods for *Yersinia enterocolitica* identification: the miniaturized biochemical tests API 20E (Biomerieux) and the MALDI-TOF MS system.

## Materials and methods

### Sample collection

Between July 2018 and July 2019, a total of 130 samples were analyzed for detection of pathogenic *Y*. *enterocolitica* as reported in Table 1.

**Table 1:** Number and type of samples tested for detection of *Yersinia enterocolitica*.

Sample		Number of samples
Raw milk	• bovine milk	44
	• water-buffalo milk	4
	• sheep milk	5
	• goat milk	4
Dairy products made from raw milk	• Mozzarella cheese • Water-buffalo Mozzarella cheese • Butter • Bovine Ricotta cheese • Ovine Ricotta cheese • Caciocavallo cheese • Scamorza cheese • Stracciatella cheese • Pecorino cheese • Bovine/ovine hard cheese • Giuncata cheese • Yogurt	32 1 1 2 1 1 6 1 1 1 1 1
Cold cuts (pork)	• Cooked ham • Mortadella • Seasoned sausage	1 2 9
Pre-packaged ready-to-eat vegetables	• Mixed salad • Carrots • Rucola • Radish sprouts	3 1 2 1
Pre-packaged fruit salad		1
Products consumed after cooking	• Beef and pork mixed ground meat	2
	• Chicken burger	1
	• Chicken/turkey cordon bleu	1
**Total**		130

Food products representing a possible risk for the consumer were chosen: in particular, raw dairy products, pork cold cuts and ready‐to‐use vegetables. Furthermore, some products that should be eaten after cooking but for which insufficient cooking can occur, were analyzed. Most sampled foodstuffs were typical local products, such as Mozzarella cheese made from raw milk, in order to establish its safety for the consumer.

Furthermore, raw bulk-tank milk samples, susceptible to faecal contamination during milking, were also examined, since pathogenic *Y*. *enterocolitica* O:9 was frequently isolated by the Experimental Zooprophylactic Institute of Apulia and Basilicata from ruminants inhabiting these two regions and which showed false-positive serological reactions to *Brucella* spp.

Most samples (118 products) consisted of official samples sent to the “food microbiology” laboratory of the Experimental Zooprophylactic Institute of Apulia and Basilicata for official microbiological control in accordance with the Integrated Regional Control Plan of Apulia and Basilicata region and were also tested for the purpose of this study. They were collected at retail outlets or from the manufacturer-suppliers, at the end of the manufacturing process: in particular, 98 raw milk/dairy product samples from cheese factories, 5 cold cuts from retail butchers and 5 pre-packaged ready-to-eat vegetables, 1 pre-packaged fruit salad, 4 meat preparations and 5 cold cuts at supermarkets.

In addition, 2 cold cuts and 2 pre-packaged mixed vegetable salads were purposely purchased at retail outlets.

Moreover, 8 individual milk samples were taken from as many bovines reared in a *Brucella*-free cattle farm which reacted positive to the brucellosis official serological tests in order to search simultaneously *Brucella* spp. and *Y*. *enterocolitica*. The animals were sacrificed in a slaughterhouse in accordance with the national legislation and epidemiological and diagnostic examinations were performed.

Most samples were collected in two provinces, Foggia (61/130; 47%) and Barletta-Andria-Trani (39/130; 30%).

With regard to dairy products, some of them were analyzed immediately after sampling while others were frozen at -20°C and analyzed later, since freezing/thawing process does not kill *Y*. *enterocolitica* [52].

### Isolation and identification of *Y*. *enterocolitica*

Samples were examined using both molecular and culture methods simultaneously, starting from the same enrichment in non-selective PSB broth, according to ISO 18867:2015 Annex B-Method 1 [53] and ISO 10273:2017 [54] respectively. However, direct plating from PSB broth onto CIN agar plates and the selective enrichment phase in supplemented ITC broth were avoided.

The molecular method allowed the detection of pathogenic *Y*. *enterocolitica* by amplification of the *ail* gene by Real-Time PCR in combination with a heterologous Internal Amplification Control (IAC) based on the plasmid pUC 19.

The confirmation of the suspect colonies isolated on CIN agar was carried out by using both the miniaturized biochemical tests API 20E (Biomerieux) and the MALDI-TOF MS system. The subsequent biotyping and serotyping of the confirmed colonies were performed according to ISO 10273:2017.

#### MALDI-TOF MS procedure

The isolated strains were streaked onto Tryptone Soya agar and incubated at 30°C for 24h and analyzed in duplicate. A fresh single colony was smeared onto one out of 96 spots of the target plate (Bruker Daltonics, Germany) and left to dry at room temperature. Subsequently, the sample spots were overlaid with 1 μl of α-cyano-4-hydroxycinnamic acid (HCCA, Bruker Daltonics, Germany) (10 mg/mL) and the obtained mixture was left to dry and crystallize at room temperature. Each spot of the target plate was hit with a pulsed nitrogen laser beam operating at 337 nm, with a frequency equal to 60 Hz. After laser shot, the gas phase ions obtained were accelerated in the flight tube by an acceleration voltage with optimized values for the mass range under study. Two hundred and forty individual laser shots were added for each spectrum. The instrument was calibrated in the broad molecular weight range between 2 and 20 kDa using Bruker Bacterial Test Standard (BTS, Bruker Daltonics, Germany), an extract of the *Escherichia coli* DH5α strain, with the addition of two proteins (RNase A of 13,683.2 Da and myoglobin of 16,952.3 Da).

The target plate was loaded into the MALDI-TOF instrument Microflex LT/SH^TM^ (Bruker Daltonics, Germany) which was operated in linear positive mode covering a mass to charge ratio (m/z) between 2000 and 20,000 and the generated mass spectra were acquired by the FlexControl 3.4 software and processed by MBT Compass 4.1.70.20 software (Bruker Daltonics, Germany). The spectra were compared to the reference database MBT Compass library v 7.0.0.0 for bacterial identification. A log (score) value ≥ 2.00 was considered an excellent probability for identification at the species level. The reference database included more than 7000 Main Spectrum Profiles (MSPs), 17 of them referred to *Y*. *enterocolitica* species.

#### Genotypic characterization

Moreover, genotypic characterization was performed only on *Yersinia* strains identified by API 20 E and MALDI TOF MS discordantly. The genomic DNA was extracted from 7 *Yersinia* isolates using DNeasy Blood and Tissue Kit (Qiagen, Hilden, Germany), according to the manufacturer’s protocol. DNA quality and concentrations were estimated by Qubit Fluorometer using Qubit dsDNA HS Assay (Thermo Fisher Scientific). A portion of 16S rRNA and *gyrB* genes were amplified by universal primers as reported by Bonerba *et al*. [55] and Yamamoto and Harayama [56], respectively. Amplification reactions were performed in a total volume of 25 μL containing 12.5 μL 1X Taq polymerase buffer, 0.5 mM of each primer, 2.5 mM of each dNTPs, 0.5 unit of HotStarTaq Plus DNA Polymerase (Qiagen) and approximately 10ng of DNA sample. The amplification process for both polymerase chain reaction (PCR) was carried out in a thermal cycle as reported by Bonerba *et al*. [55] and Yamamoto and Harayama [56]. The amplicons were visualized by QIAxcel Advanced system (Qiagen), an automates electrophoresis analysis and then they were purified using the exonuclease and phosphates enzymes. Both strands were amplified and sequenced on ABI Prism 3130 Genetic Analyzer (Applied Biosystem ThermoScientific inc.) as described by Bianco *et al*. [57]. All sequences were analysed using Bionumerics v7.5 software (Applied Maths, Belgium).

The libraries were prepared using Illumina technologies and the reads were assembled as reported by Bianco *et al*. [58]. The draft genome of each isolate was submitted in ribosomal multilocus sequence typing (rMLST) database (https://pubmlst.org/species-id) to verify taxonomic classification.

## Results

The 130 samples analyzed according to ISO 18867:2015 for detection of *ail* gene were negative. Conversely, the culture method allowed to isolate suspicious colonies from 28 samples. All strains were isolated on CIN agar plates after potassium hydroxide (KOH) treatment.

**Fig 1:**
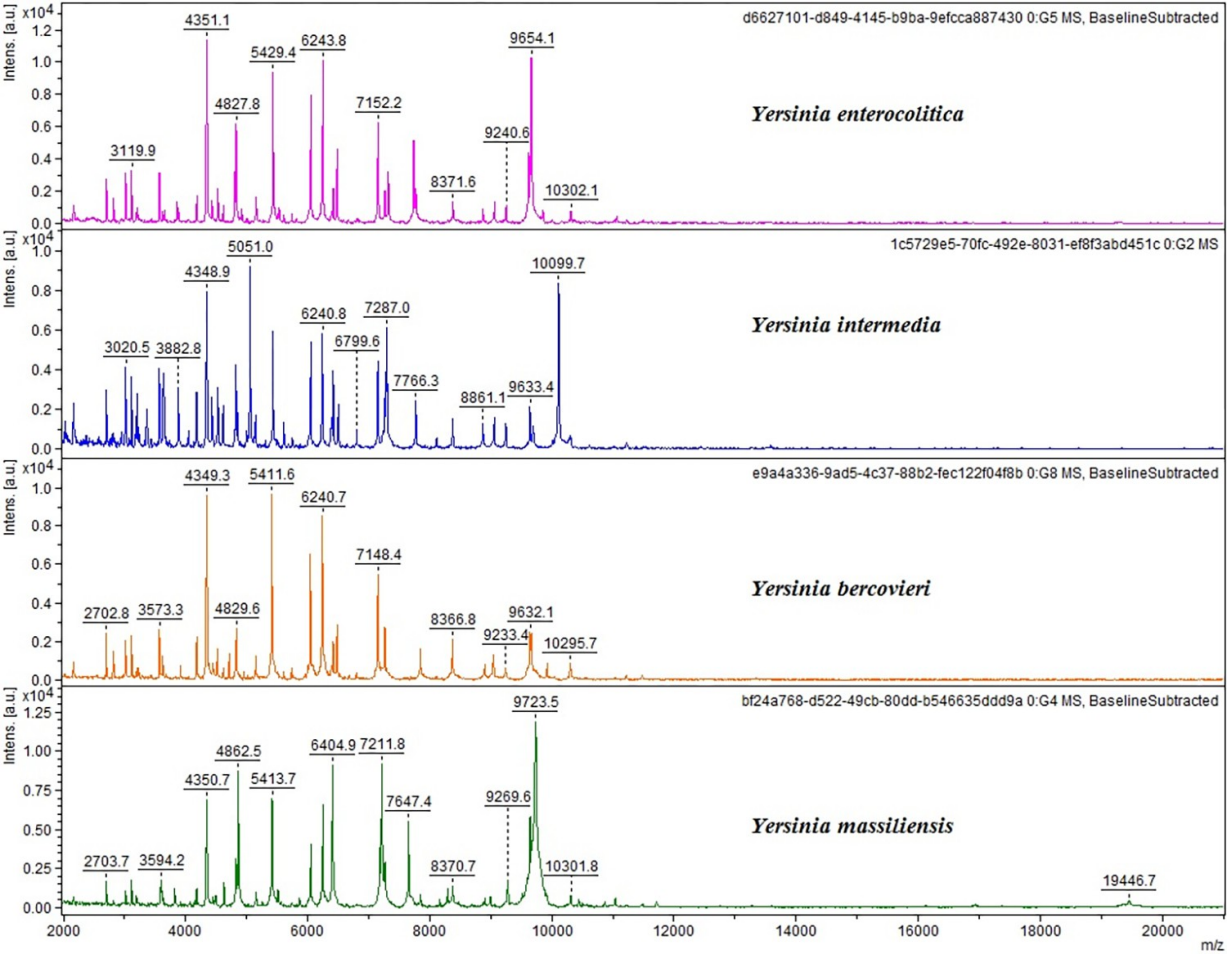
MALDI-TOF mass spectra of four *Yersinia* species in the mass range of 2 to 20 kDa: *Y enterocolitica*, *Y*. *intermedia*, *Y*. *bercovieri*, *Y massiliensis*. The mass spectra were obtained using the FlexAnalysis software (v. 3.4, Bruker Daltonik GmbH, Bremen, Germany), performing baseline corrected, smoothed and normalized analyses.

The API 20E system and the MALDI-TOF MS technique identified 20 *Y*. *enterocolitica* and 1 *Y*. *intermedia* in a concordant way. The remaining 7 strains were identified as *Y*. *enterocolitica* by the API 20E system, while the MALDI-TOF MS recognized 4 *Y*. *intermedia*, 1 *Y*. *bercovieri* and 2 *Y*. *massiliensis*. The typical mass spectra acquired from some isolates belonging to the different *Yersinia* species detected are illustrated in Fig 1.

Sequences of 16S rRNA and *gyrB* genes obtained from the 7 discordant strains were predicted as *Y*. *intermedia*, with a percentage of identity of 100%, independently from the gene analyzed. The predicted taxa provided by rMLST database were 4 *Y*. *intermedia*, 1 *Y*. *bercovieri* and 2 *Y*. *massiliensis*, in accordance with the MALDI-TOF MS’ identification. Sequencing data are available at NCBI BioProject with accession number PRJNA770150.

*Yersinia enterocolitica* strains were isolated from dairy products made from raw milk (6/49), beef and pork mixed ground meat (1/2), raw cow milk (12/44), and raw goat milk (1/4). No *Yersinia enterocolitica* were detected in the individual milk samples collected from cattle which showed serologically false-positive reactions for *Brucella* spp., although pathogenic *Y*. *enterocolitica* O:9 biotype 2 strains, harboring the *ail* gene, were isolated from their intestinal tract contents.

All 20 *Y*. *enterocolitica* strains (Prevalence = 0.15 with I.C. 95% = ± 0.06) belonged to biotype 1A. Serotypes identified were O:5 (7/20), O:8 (4/20) and O:27 (1/20); 8 strains did not show any agglutination with available antisera (O:3, O:5, O:8, O:9 and O:27). More information is detailed in Table 2.

**Table 2:** Results on identification and typing of Yersinia isolates: Comparison among different analytical methodologies.

Sample	MALDI-TOF species identification	API 20E species identification	Biotype	Serotype	*ail* gene	Sanger sequencing *16S*	Sanger sequencing *gyrB*	WGS rMLST (BioSample number)
Mozzarella cheese	*Y*. *enterocolitica*	*Y*. *enterocolitica*	1A	O:5	-			
Raw cow milk	*Y*. *enterocolitica*	*Y*. *enterocolitica*	1A	n.t.	-			
Scamorza cheese	*Y*. *enterocolitica*	*Y*. *enterocolitica*	1A	O:8	-			
Raw cow milk	*Y*. *enterocolitica*	*Y*. *enterocolitica*	1A	O:5	-			
Mozzarella cheese	*Y*. *enterocolitica*	*Y*. *enterocolitica*	1A	O:8	-			
Raw cow milk	*Y*. *enterocolitica*	*Y*. *enterocolitica*	1A	O:5	-			
Beef and pork mixed ground meat	*Y*. *enterocolitica*	*Y*. *enterocolitica*	1A	O:5	-			
Raw goat milk	*Y*. *enterocolitica*	*Y*. *enterocolitica*	1A	n.t.	-			
Mozzarella cheese	*Y*. *enterocolitica*	*Y*. *enterocolitica*	1A	O:8	-			
Mozzarella cheese	*Y*. *enterocolitica*	*Y*. *enterocolitica*	1A	n.t.	-			
Raw cow milk	*Y*. *enterocolitica*	*Y*. *enterocolitica*	1A	O:5	-			
Raw cow milk	*Y*. *enterocolitica*	*Y*. *enterocolitica*	1A	n.t.	-			
Raw cow milk	*Y*. *enterocolitica*	*Y*. *enterocolitica*	1A	n.t.	-			
Raw cow milk	*Y*. *enterocolitica*	*Y*. *enterocolitica*	1A	O:8	-			
Raw cow milk	*Y*. *enterocolitica*	*Y*. *enterocolitica*	1A	n.t.	-			
Raw cow milk	*Y*. *enterocolitica*	*Y*. *enterocolitica*	1A	O:5	-			
Raw cow milk	*Y*. *enterocolitica*	*Y*. *enterocolitica*	1A	O:5	-			
Raw cow milk	*Y*. *enterocolitica*	*Y*. *enterocolitica*	1A	O:27	-			
Raw cow milk	*Y*. *enterocolitica*	*Y*. *enterocolitica*	1A	n.t.	-			
Mozzarella cheese	*Y*. *enterocolitica*	*Y*. *enterocolitica*	1A	n.t.	-			
Mozzarella cheese	*Y*. *intermedia*	*Y*. *enterocolitica*		n.t.	-	*Y*. *intermedia*	*Y*. *intermedia*	*Y*. *intermedia* (SAMN22081163)
Mozzarella cheese	*Y*. *intermedia*	*Y*. *enterocolitica*		n.t.	-	*Y*. *intermedia*	*Y*. *intermedia*	*Y*. *intermedia* (SAMN22081164)
Raw cow milk	*Y*. *intermedia*	*Y*. *enterocolitica*		n.t.	-	*Y*. *intermedia*	*Y*. *intermedia*	*Y*. *intermedia* (SAMN22081166)
Raw cow milk	*Y*. *intermedia*	*Y*. *enterocolitica*		n.t.	-	*Y*. *intermedia*	*Y*. *intermedia*	*Y*. *intermedia* (SAMN22081167)
Cooked ham	*Y*. *intermedia*	*Y*. *intermedia*		n.t.	-			
Mixed salad	*Y*. *bercovieri*	*Y*. *enterocolitica*	3B	O:5	-	*Y*. *intermedia*	*Y*. *intermedia*	*Y*. *bercovieri* (SAMN22081165)
Raw buffalo milk	*Y*. *massiliensis*	*Y*. *enterocolitica*		n.t.	-	*Y*. *intermedia*	*Y*. *intermedia*	*Y*. *massiliensis* (SAMN22081168)
Carrots	*Y*. *massiliensis*	*Y*. *enterocolitica*		O:27	-	*Y*. *intermedia*	*Y*. *intermedia*	*Y*. *massiliensis* (SAMN22081169)

n.t.: not typeable.

## Discussion

With regard to the detection procedure, the culture method according to ISO 10273:2017 with some changes useful to reduce the execution time was effective for isolation of 20 *Y*. *enterocolitica* strains (28 *Yersinia* spp. strains). In particular, preventive treatment with KOH before plating onto CIN agar was indispensable to observe pure characteristic colonies, without background flora.

The two methods for species identification (API 20E and MALDI-TOF MS) showed discordant results. Unlike the API 20E system, the MALDI-TOF MS technique was able to correctly identify 7 *Yersinia* spp. strains, as confirmed by rMSLT results, used as a reference method. It is well known that biochemical identification system often fails to ensure reliable identification at species level for *Y*. *enterocolitica* [46, 47, 59, 60]. Moreover, the API 20E database does not include all *Yersinia* species but it lists only 6 of the currently known 18 species, i.e. *Y*. *enterocolitica*, *Y*. *pseudotubercolosis*, *Y*. *pestis*, *Y*. *intermedia*, *Y*. *kristensenii*, *Y*. *frederiksenii*, therefore *Y*. *bercovieri* and *Y*. *massiliensis* cannot be identified. In the present study, other Yersinia species have been misidentified as *Y*. *enterocolitica* by API 20E. However, *Y*. *bercovieri* strain has been identified by API 20E and subsequent biotyping as *Y*. *enterocolitica* O:5 biotype 3, in accordance with the original nomenclature [61], whereas *Y*. *massiliensis* strains have been recognized by API 20E as *Y*. *enterocolitica* with non-excellent identification percentage (% ID = 81.5).

Regarding the remaining discordant isolates, *Y*. *intermedia* is easily differentiated from *Y*. *enterocolitica* by positive reaction for L-rhamnose and melibiose, while *Y*. *enterocolitica* consists of L-rhamnose and melibiose negative strains. Therefore, these biochemical tests have been repeated by using the appropriate liquid media in test tubes. Two isolates showed negative reaction, so misidentification derives from their unusual biochemical behavior. The other two isolates, instead, showed positive reaction, in contrast with the API 20E results. It should be considered that differentiation by biochemical tests is usually based on a limited set of strains, which can generate contradictory results [59].

In general, MALDI-TOF MS technique appeared as a suitable alternative method for species identification because it showed better discriminatory capacity, allowing to reveal the misidentifications provided by the API 20E. Even if the API system did not produce false negative results in *Y*. *enterocolitica* identification, it generated false positive results when other Yersinia species occurred. This misidentification can lead to an overestimation of the prevalence of the microorganism. However, the isolates misidentified as *Y*. *enterocolitica* were nonpathogenic strains, therefore there was no possibility of assessing the food product unsafe for the consumer.

The use of MALDI-TOF MS showed several advantages compared to biochemical tests: the ease of procedure, the possibility of automation, the rapid data acquisition, the minimal costs of consumables and the high-throughput analysis (up to 96 samples analyzed simultaneously), as already stated by other authors [51, 62, 63]. Furthermore, the miniaturized biochemical identification system provided not always reliable results and could suffer from a certain subjectivity in the colorimetric evaluation. Therefore, it would be desirable to use, after appropriate validation, MALDI-TOF MS in routine as method to support official food controls, especially since even biomolecular methods such as Sanger sequencing of 16S rRNA and *gyrB* genes failed to correctly identify the microorganism. The main limitation of MALDI-TOF MS technology is that identification of the unknown test organism is only possible if the reference database contains the specific MSPs: in fact, low number of spectral representations in the library could lead to no identification [64], therefore database should undergo a continuous implementation and updating in order to generate reliable results. The completeness of reference library is of crucial importance. Since differences in the proteins produced by different strains within the same species can occur [65], the database should contain several reference spectra for multiple strains for each species of interest [66]. Moreover, if low Confidence Value or non-correspondent outcomes between duplicates occur, further confirmation methods, such as PCR or sequencing, could be necessary.

The isolation of non-pathogenic strains of *Y*. *enterocolitica* biotype 1A with a non-negligible prevalence (P = 0.15 with 95% CI = ± 0.06) is not surprising, since it is a ubiquitous and widespread microorganism. The detection of *Y*. *enterocolitica* in raw bulk-tank milk from different species (P = 23%) agrees with results reported by Ahmed *et al*. [13] (P = 22%) and Hadef *et al*. [67] (P = 17%) while it differs from other authors’ reports. In fact, Alavi *et al*. [39], Bonardi *et al*. [41], Darwish *et al*. [42] and Jamali *et al*. [43] recorded lower prevalence values (9%, 3.1%, 11% and 4.3%, respectively). In the above-mentioned works, both pathogenic and non-pathogenic *Y*. *enterocolitica* strains were isolated, although the non-pathogenic strains outnumbered the pathogenic ones; in the present study, instead, only non-pathogenic strains have been found, despite evidence of circulation of pathogenic *Y*. *enterocolitica* in dairy farms located in the investigated regions as shown by the Experimental Zooprophylactic Institute of Apulia and Basilicata’ activity. Moreover, the diagnostic tests carried out on bovines serologically positive for brucellosis confirmed the absence of *Brucella* spp., while pathogenic *Y*. *enterocolitica* O:9 biotype 2 strains were isolated from their faeces, but not from milk samples. Hence, the bacterium is not able to contaminate the milk if the correct milking practices are followed, even in the presence of gastro-intestinal infection. In the present study, sometimes both raw bulk-tank milk and dairy products made from it were analyzed simultaneously: in four cases, non-pathogenic *Y*. *enterocolitica* was detected only in raw milk but not in its relative derived product. This could suggest the idea that the cheese manufacturing process, even if involves heat treatment at temperature lower than pasteurization, can be equally effective in eliminating the microorganism. However, non-pathogenic *Y*. *enterocolitica* strains have been isolated from dairy products made from raw milk, but in these cases the corresponding raw bulk-tank milk were not analyzed. Although it is more reasonable to assume that this is due to a subsequent post-process contamination, the presence of the bacterium in the starting raw milk and its survival during the production process cannot be excluded.

In conclusion, the lack of detection of pathogenic *Y*. *enterocolitica* in foodstuffs produced and/or marketed in the investigated territories suggests a low risk for the consumer. However, the isolation of non-pathogenic strains on ready-to-eat foods, presumably related to an improper application of good hygiene practices during processing, entails the need to pay attention to these foodstuffs anyway, especially because the circulation of pathogenic strains among farms in Apulia and Basilicata regions has been demonstrated. Moreover, the small number of samples examined in the present work, insufficient for some food categories such as meat and vegetable products, does not allow to exclude the presence of pathogenic strains at all, but suggests the opportunity to conduct further investigations.
